# Pharmaceutical “New Prior Knowledge”: Twenty-First Century Assurance of Therapeutic Equivalence

**DOI:** 10.1208/s12249-019-1347-6

**Published:** 2019-03-12

**Authors:** Ajaz S. Hussain, Vadim J. Gurvich, Kenneth Morris

**Affiliations:** 1 The National Institute of Pharmaceutical Technology and Education (NIPTE), Inc., Minneapolis, Minnesota 55414 USA; 2 Arnold and Marie Schwartz College of Pharmacy and Health Sciences, 75 DeKalb Avenue, Room WL 313, Brooklyn, New York 11201 USA; 3 Institute for Therapeutics Discovery and Development, College of Pharmacy, University of Minnesota, Minneapolis, Minnesota USA; 4 Lachman Institute for Pharmaceutical Analysis, Long Island University, Brooklyn Campus, Brooklyn, New York 11201 USA

**Keywords:** new prior knowledge, generic drug prices, mometasone furoate, enoxaparin, levothyroxine

## Abstract

Facilitating utility of prior knowledge to accelerate evidence-based new drug development is a focus of several communities of knowledge, such as clinical pharmacology. For example, progress has been made *via* modeling and simulation of pharmacokinetic and pharmacodynamic relationships in the more effective use of “End of Phase 2” regulatory meetings for a New Drug Application (NDA). Facilitating utility of prior “Chemistry, Manufacturing, and Controls” (CMC) knowledge to accelerate new drug development and regulatory review process is also a topic of significant interest. This paper focuses on facilitating the utility of prior pharmaceutical formulation knowledge to accelerate drug product development and regulatory review of generic and biosimilar products. This knowledge is described as New Prior Knowledge (NPK) because research is often needed to fill ontological (*i*.*e*., the domain of connectivity between concepts and phenomena), epistemological (*i*.*e*., distinguishing knowledge or justified belief from the opinion), and methodological gaps in information derived a decade or so ago. The corporate economic advantages of such knowledge are derived, in part, when significant portions remain a trade secret. The proposed NPK seeks to generate knowledge about critical aspects of pharmaceutical quality and failure modes to place it in the public domain and to facilitate accelerated and more confident development and regulatory review of generic products. The paradoxical combination of “new” and “prior knowledge” is chosen deliberately to highlight both a distinction from proprietary and trade secret information and to acknowledge certain historical dogmas inherent in the current practices. Considerations for operationalizing NPK are also summarized.

## INTRODUCTION

The current AAPS PharmSciTech Theme Issue, “Team Science and Education for Pharmaceuticals: the NIPTE Model,” highlights various US FDA-sponsored research projects conducted at the National Institute for Pharmaceutical Technology and Education (NIPTE). NIPTE is a unique 501(c)(1)(3) non-profit academic organization fostering multi-university collaboration to fill critical gaps in pharmaceutical technology research and education. An unmet critical societal need that NIPTE seeks to address is ensuring the availability of affordable medicines with a high assurance of quality (1). This paper discusses how information generated *via* collaborative public research can be transformed into the valuable knowledge needed to address the unmet need of availability of affordable medicines with the high level of assurance expected by the American public.

Increased competition through the availability of generic and biosimilar medicines is the preferred way to improve the affordability of, and access to, medicines in the USA. This competitive mechanism relies on prior knowledge to reduce the cost of development and regulatory approval, as well as the cost of goods while improving manufacturing efficiency and reducing the need for detailing and marketing. Thus, pharmaceutical “new prior knowledge,” or NPK, can significantly improve the reliability and efficiency of this process and increase the speed to market for generic drugs (2).

## PHARMACEUTICAL “NEW PRIOR KNOWLEDGE” (NPK)

The paradoxical combination of “new” and “prior knowledge” is intended to distinguish NPK from traditional and proprietary pharmaceutical “prior knowledge.” Critically, it is also intended to draw attention to the paradoxical state of collective inattention to the root cause of challenges to the availability of affordable medicines with a high assurance of quality.

As the ancient adage goes, “nothing new under the Sun.” Science builds on prior knowledge. Balancing policy incentives (*e*.*g*., intellectual property, data and market exclusivity) for generation of new knowledge and encouraging the availability, diffusion, and reuse of prior knowledge can have a significant impact on the rate of economic growth and development.

The use of prior knowledge is integral to new drug development. For example, objective, quantitative, and generalizable knowledge is the basis for clinical pharmacology considerations in Phase II clinical trials. However, the bulk of the prior knowledge available to generic sponsors is on the molecule itself. Product development knowledge is limited for significant portions of prior pharmaceutical information, such as pharmaceutical formulations and their development, characterization, and manufacture. These are often trade secrets that may remain proprietary indefinitely. The available pharmaceutical information in the public domain tends to be empirical, often incomplete and is difficult to generalize. Beyond minimal compendial testing, little pharmaceutical knowledge about the quality of marketed products is available to the public. The current ontological informatics infrastructure for pharmaceutical materials, formulations, and manufacturing processes is diverse and fragmented, with weak epistemology and assorted methodologies. Filling these gaps would require significant effort. However, it is the only rational way to address the urgent national need to enable knowledge and good manufacturing practices to be modeled and captured into tools to support the product lifecycle management (3–5). New prior knowledge relevant for pharmaceutical product development is, therefore, the aggregated information, in the context of likely failure modes, that link the characteristics of the drug substance to the physical-chemical to product performance, therapeutic equivalence, and demands of creating a manufacturable quality dosage form.

**Fig. 1:**
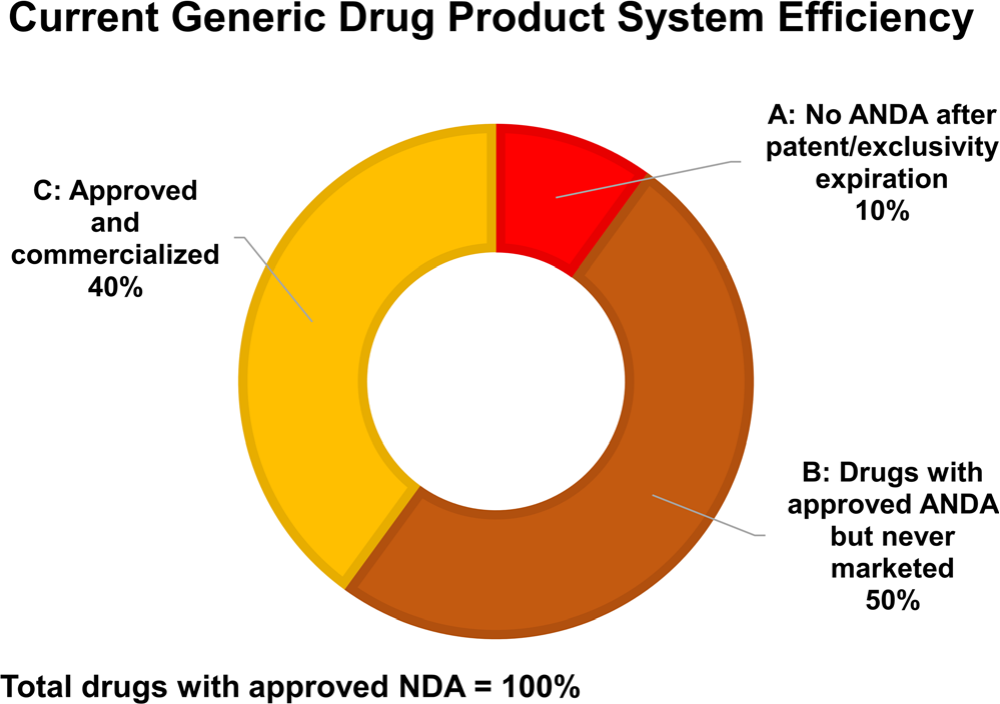
Current challenges in approval of ANDA’s. **A** For 10% of all FDA-approved NDA’s (100%), no ANDA has been submitted despite expired patents and exclusivities (6). **B** Quoting an FDA issued Federal Register notice—“The drugs described in more than half of all FDA-approved ANDAs are never marketed, marketed only after a substantial delay after approval, or marketed only intermittently. Such failures to market contribute to drug shortages and hinder consumer access to approved products” (7). **C** Percentage of approved and commercialized ANDA’s

To illustrate the need, consider that, according to the US FDA, Abbreviated New Drug Applications (ANDA) are not submitted for generic versions of about 10% of approved brand drugs despite the expiration of patents and exclusivities. Additionally, over 50% of approved generic drugs are either never marketed, or reach the market with a substantial delay after approval, or marketed only intermittently (Fig. 1) (6,7). A recent analysis of FDA’s 2017 and 2018 lists of first generics revealed that of the more than 1600 approved generic drug applications in 2017, more than 700, or about 43%, still were not on the market as of early January 2019. Many administrative reasons are generally cited for delays and lack of availability of these products (8). Scale-up, process validation, and repeatability can pose challenges, and this aspect is often not recognized in congressional deliberations and media reports. These challenges can be related to rush to file, the establishment of regulatory specifications following bio-study, and residual uncertainty as different lots of raw materials are encountered. All of these technical reasons can be considered as gaps in prior knowledge. In a typical pharmaceutical business model, incentivized by 180-day exclusivity to “file first,” some sources of variance (*e*.*g*., lot-to-lot variability of raw materials) and patient-, process-, and product-related failure modes maybe not in the scope of a development plan. A review of recent US FDA Warning Letters points to breaches in the assurance of data integrity, failures in process validation, supplier controls, testing of incoming raw materials, and need to improve process monitoring to ensure the process remains under control as areas of concern (9).

In this paper, three specific examples are selected, based on the experience of the authors, to identify and describe the essential practical features of NPK in the context of the availability of generics (10). Additionally, the way NPK contributes to the assurance of quality and therapeutic equivalence is discussed. It is posited that the assurance patients and their care providers expect in therapeutic equivalence will continue to increase alongside the growing regulatory emphasis on real-world evidence, personalized medicines, and patient-centric new drug development as mandated by the US twenty-first Century Cures Act (Cures Act) enacted in December 2016 (11). Practical steps that NIPTE, and perhaps other communities of pharmaceutical knowledge, can take to establish formal systems for generating, curating, and disseminating NPK are briefly summarized.

## EXAMPLES OF PHARMACEUTICAL NPK

In theory, US FDA approval of generic and licensed interchangeable biosimilar medicinal products (12,13) eliminates the need for the cost of marketing and detailing. Therefore, facilitating the availability of these products *via* an abbreviated development and approval pathway (*e*.*g*., by minimizing costly clinical trials) and promoting continual improvement to reduce variance and increase manufacturing efficiency should be a significant goal of generating, curating, and disseminating pharmaceutical NPK. Unfortunately, significant gaps persist (14,15). Within this context, three examples are selected to identify and describe the practical features of pharmaceutical NPK. The first two examples pertain to improving the efficiency of the current abbreviated generic development and regulatory pathways. The third example seeks to improve awareness of how inadequate NPK can delay or limit continual improvement and erode assurance in therapeutic equivalence.

## GENERIC (USA) AND BIOSIMILAR (EU) ENOXAPARIN

Enoxaparin (low molecular weight heparin) is a complex mixture of oligosaccharides formulated as a simple aqueous solution for injection. For therapeutic equivalence assessment of generic enoxaparin, the demonstration of pharmaceutical equivalence, rather than bioequivalence, is the primary challenge. That involves analytical characterization to demonstrate that generic products contain the same active ingredient as the reference product and the comparability of purity and quality to ensure generic enoxaparin would not pose a higher safety risk than its reference product. In 2010, the US FDA approved the first generic enoxaparin product without clinical safety or efficacy data under the Abbreviated New Drug Application (ANDA) pathway. At the same time, the European Medical Agency decided to regulate follow-on enoxaparin products within its biosimilar regulatory pathway. The US FDA described its approach to approval in a milestone publication noting that this approval “represents a major development in US regulatory science and policy that will likely affect several other complex drug products” (16).

The FDA approval of generic enoxaparin based on extensive analytical characterization, significantly exceeding the level of testing in a typical compendial monograph, illustrates its value in confirming, with confidence, the assessment of identity, comparable purity, quality (an essential consideration for pharmaceutical equivalence), and the development of control strategy for the manufacturing process. The approval of the first generic enoxaparin represents an example of privately (in this case by Sandoz-Momenta) generated NPK (10).

The generic enoxaparin approval signifies the US FDA’s institutional confidence in its Chemistry, Manufacturing, and Control, or CMC, review expertise. Moreover, recognizing that information is not knowledge, the enoxaparin example highlights differences between the US and the EU in regulatory policy preference in the context of complexity and uncertainty, *i*.*e*., sameness *vs*. similarity. In the EU, follow-on enoxaparin is considered a biosimilar for which interchangeability is not officially endorsed in the EU regulatory system. The example also points to the competitive advantage of private NPK for achieving first to market status and benefiting from limited competition.

Public NPK is preferable to support the timely (*i*.*e*., immediately after the expiration of the patent term and any exclusivity period) availability of generics and to maximize competition. It should be noted that since 2007, the rate of new generic entrants to the market has markedly slowed and the number of firms competing for individual drugs has fallen, while generic drug prices have risen by a statistically significant margin (17).

## FIRST GENERIC MOMETASONE FUROATE NASAL SPRAY

The generic applicant, Apotex, developed its Mometasone Furoate nasal spray product before FDA guidance was established and used a different polymorph of the drug so as not to infringe on reference product’s unexpired patent. Apotex submitted its ANDA (with paragraph IV certification) to the US FDA in December 2008. In 2016, the US FDA approved the first generic after about 8 years of multiple review cycles. The approval was finalized several years after the expiration of the reference product’s patent term and a formal scientific dispute resolution on the FDA’s requirement for repeating a clinical trial (18).

A “weight of evidence” approach is used to establish therapeutic equivalence for locally acting drug products such as nasal sprays. The evidence includes sameness in device and formulation and equivalent *in vitro* performance of several attributes such as Single Actuation Content, Droplet Size Distribution by Laser Diffraction, Spray Pattern, Plume Geometry, Priming and Repriming, and Drug in Small Particles/Droplets. Furthermore, evidence of pharmacokinetic (systemic absorption) bioequivalence and a clinical bioequivalence trial—a randomized, double-blind, three-arm, placebo-controlled, parallel group of subjects with seasonal allergic rhinitis (self-scoring symptoms of a runny nose, sneezing, nasal itching, and congestion)—is also required. The clinical endpoint study was recommended because the FDA had concluded that adequate characterization of drug particle size distribution (PSD) in aerosols and sprays was not feasible (19).

The applicant had successfully conducted the proposed clinical trial to establish clinical bioequivalence. However, the tested formulation failed to meet equivalence criteria for systemic pharmacokinetic exposure due to differences in particle size. Changing the drug substance to ensure *in vitro* equivalence of PSD resolved the noted difference in systemic pharmacokinetic exposure (which is the fraction of the drug in the systemic circulation and therefore, not at the site of action; *i*.*e*., nasal epithelium). The scientific dispute between the applicant and FDA was focused on the requirement to repeat the expensive and time-consuming clinical trial which, in the first instance, concluded equivalence despite the noted difference in particle size in the test and reference product. The residual uncertainty underpinning FDA requirement to repeat the clinical trial was not just particle size difference but the stability of crystal habit (associated with the polymorphic form selected) of the drug substance used in the generic product. Instability of particle shape in the product could pose a risk by the formation of needle-shaped particles; as nose-bleed is a known and significant adverse effect (10,18).

Resolution of the dispute was achieved in reaching an agreement on what is the residual uncertainty in the context of a patient-related failure mode and establishing scientific multi-disciplinary consensus on the “weight-of-evidence” in the context of the failure mode, risk assessment, and measurement system to monitor and control it, instead of a one-time clinical trial (10). In this case, the appropriate application of morphologically directed Raman Spectroscopy was accepted for assessment of the equivalence of particle size and shape and this, “sets a precedent to accept an in vitro approach in lieu of a clinical endpoint bioequivalence study,” quoting FDA (18).

In contrast to the enoxaparin case example, where the US FDA approved an ANDA based on (extensive) analytical characterization, in the case of the nasal spray application, it sought certainty in a clinical trial. Why? The safety concern (*e*.*g*., immunogenicity) with enoxaparin was derived from purity and impurity differences in a solution, whereas for the nasal spray, it was related to a known (for reference product) and significant adverse effect, nose bleeding. The immunogenicity concern is more serious, as can be inferred in the EMA’s policy preference for a biosimilar regulatory path. We suggest that this difference points to, or suggests, a high (institutional) confidence in the available expertise in solution analytical chemistry at the US FDA. The need to build expertise in solid-state material science, physical chemistry, physical pharmacy, and pharmaceutical engineering has been recognized previously (14,15). Information and knowledge of solid-state material science and analytical characterization in the context of patient-related failure modes are a significant need that NPK efforts need to address.

In the nasal spray example, conducting the recommended clinical study was not considered a problem by both the applicant and FDA. In the proposed NPK context, automatic reliance on clinical trials should be raised as a research question. The need to repeat the clinical study was objectionable, a problem that needed resolution, even though evidence of its “bluntness” or inability to distinguish between particle size differences existed. Furthermore, some relevant information about the precedent-setting decisions was available within the FDA for many years. Over a decade ago, a US FDA laboratory, Division of Pharmaceutical Analysis, published a paper entitled “Raman Chemical Imaging for Ingredient-specific Particle Size Characterization of Aqueous Suspension Nasal Spray Formulations: A Progress Report” (20). Clearly, during this review process, the FDA had access to know-how on state-of-the-art analytical tools, such as imaging tools and methodologies, and this publication provides information that seeks to inform the community at large about their learning on how to measure ingredient-specific particle size in nasal spray formulations. These observations point to a need for NPK efforts to bridge the gaps between research to policy and policy to practice. Sharing information by merely placing it in the public domain is often insufficient until there is a need for the information to solve a problem, and with application in specific context, information is transformed into knowledge.

Scientific disputes and debates identify the need for NPK, and the experience gained from the nasal spray dispute resolution process provides valuable insights into the process for generating NPK. Problem identification and description in the context of patient and public health needs and expectations must be an essential element of NPK. In generating NPK, significant attention must be placed on integrating cross-domain information considering the meaning different decision-makers and stakeholders will make within their specific context.

In drug product development and regulatory review, the familiar context shared by all professional decision-makers should be preventing harm to patients by reducing residual uncertainty concerning patient-related failure modes. This context can and should be an essential integrating thread or element across multi-disciplinary information, and such integration should facilitate similar sensemaking among multiple stakeholders and decision-makers.

## THERAPEUTIC EQUIVALENCE OF LEVOTHYROXINE TABLETS

For generic and biosimilar medicinal products, pharmaceutical equivalence can be the “Achilles heel” in concluding sameness, biosimilarity, and interchangeable biosimilarity. “Achilles heel” is a metaphor for a weakness in apparent strength or confidence. Shifting the mindset from “pivotal” (one-time) bioequivalence or clinical trial to the integrated “weight of evidence” or “totality of evidence” for assessing and concluding therapeutic equivalence is a significant need. Education and communication, with the goal to provide patients, their care providers, and the public at large assurance in “weight of evidence” or “totality of evidence,” is an urgent need and is a more significant challenge.

Reduction of quality failures, product recalls, regulatory violations, and FDA enforcement actions (*e*.*g*., Warning Letters) and effective communication and education are essential to prevent erosion of trust. The case example of continued challenges in assuring therapeutic equivalence of levothyroxine tablets is a useful reminder of this need and provides useful insights.

The paper entitled “New Insights on Solid-State Changes in the Levothyroxine Sodium Pentahydrate during Dehydration and its Relationship to Chemical Instability” published in this Theme Issue provides a great example of NPK. It identifies a new failure mode in drug substance chemical stability. That levothyroxine remains stable at higher humidity and is unstable at low humidity raises questions on recommended stress-inducing conditions for stability testing in regulatory guidelines. Over the past several years, regulators across the globe responded to the concern, expressed by both physicians and patients, of apparent variability and therapeutic inequivalence upon switching between manufacturers of products deemed to be therapeutically equivalent. They tightened potency specification and bioequivalence goalposts. The reported failure mode and the questions it raises on the appropriateness of the recommended stress-inducing conditions for stability testing in ICH guidelines should remind and speak loudly to the community on residual uncertainty despite efforts to improve confidence in bioequivalence methodology and tightening potency specifications and it is yet another voice calling for “dire need” for NPK (21).

To place the noted “dire need” in the real-world context considers the massive backlash following the introduction of an improved formulation of levothyroxine in France and other European countries. Recently, the European Thyroid Association and Thyroid Federation International issued a joint position statement on the interchangeability of levothyroxine products in EU countries (22) which listed the following four concluding statements:

1. Several European countries have seen major health issues after a switch from one levothyroxine brand to another, as well as following the introduction of several levothyroxine formulation changes.
2. Although it is not possible to ascertain what proportion of these health issues are biologically related to the formulation change, the issues include increased prevalence of side effects as well as increased prevalence of biochemical signs of inadequate dosing and result in increased healthcare consumption and healthcare expenses.
3. Testing bioequivalence does not guarantee continued euthyroidism after a formulation change of levothyroxine.
4. In at least 3 European countries, formulation changes have been introduced by manufacturers without adequate communication with healthcare professionals and patient organizations.”

A similar experience in New Zealand is also worth noting. The introduction of a formulation deemed to be bioequivalent to the old formulation resulted in a sharp increase in the reporting of adverse reactions to this drug. Initial interpretations were attributed to a combination of factors, including mistrust of state drug-subsidizing agencies and media attention could have provoked anxiety in patients. An alternate explanation posits a change of formulation and accompanying media attention may provide patients a reason to pay attention, leading to uncertainty and anxiety, and triggering the amplification of reporting of adverse drug effects (22).

In the USA, the report on the University of Pennsylvania Health System’s efforts to promote generic substitution is also worth mentioning. They implemented a default opt-out checkbox labeled “dispense as written” in their Electronic Health Record system. If left unchecked, the generic-equivalent medication is the default (automatic). The opt-out default resulted in the overall generic prescribing rate to increase (in 7 months post-intervention) significantly for all drugs except levothyroxine. The opt-out rate for generic levothyroxine was 22.1% after the intervention, compared with less than 2% of other drugs; indicating the continued lack of confidence in generic levothyroxine among many physicians (23).

Societal concern regarding the affordability of healthcare and prescription medicines remains very high. In the USA, generic drugs are the most preferred way to improve affordability. Reports of falling adherence rate upon automatic substitution, when color and shape of tablets change (24,25), are a “canary in the coal mine,” a warning that as a sector, we are not providing the level of assurance in therapeutic equivalence for patients. At the root of the reported adherence, issue following automatic substitution may not necessarily be the color and shape of a tablet; it could be vocabulary available to patients to communicate their dissatisfaction.

The impact of changing color and shape of generic tablets on adherence should remind us of the prevailing dichotomy in the care exercised in developing a placebo for use in placebo-controlled clinical trials and a general presumption of lack of placebo effect in the real world, particularly as it pertains to the therapeutic equivalence of generics. Assurance of quality and therapeutic equivalence, in and of itself, should be considered critical to safety and efficacy patients experience in the real world. Specifically, placebo and nocebo effects are not eliminated but are only accounted for in randomized placebo-controlled clinical trials (26,27). NPK, as illustrated in (21), focused on a cause of stability quality failure, and this knowledge can contribute to preventing quality failures and associated adverse media coverage of deviations and enforcement actions, are urgently needed.

Delays in approval of complex generic drugs, drug shortages due to quality and manufacturing issues, and sub-optimal competition resulting in increasing prices of generic drug products have been a dominant concern in the USA over the past two decades. The extent of this challenge is reflected in recent media headlines such as “Fed Up With Drug Companies, Hospitals Decide to Start Their Own” (28), “With U.S. Generic Drug Market in Chaos, Indian Upstarts Rise” (29) and “Sen. Elizabeth Warren: U.S. government ought to make generic drugs” (30). Moreover, an alarming signal visible in the noise of the real world is media reports such as “If color or shape of generic pills changes, patients may stop taking them” (31).

## CONSIDERATION FOR IMPLEMENTING NPK

The system for development, regulatory approval, and commercial manufacturing of generic drugs is a complex and dynamic one which continues to evolve around “attractors” such as the incentive to “file first,” 180-day exclusivity, and the need for US FDA to approve multiple applications for the Nation to realize the benefits of competition (32). Furthermore, generic companies competing for the US market are globally distributed and vary in sizes and in the availability of resources that they can use for their development projects. NPK projects that NIPTE and other academic or non-academic programs could consider would need to focus on generating information and knowledge that supplement what is available in compendial monographs. That will help facilitate industry and FDA efforts to improve quality of regulatory submissions and minimize adverse regulatory outcomes such as multiple review cycles, “Complete Response Letters,” “Warning Letters,” and product recalls. When necessary, as illustrated with a levothyroxine case example, NPK projects can address challenges confronting already marketed products.

To provide “totality of evidence” and to minimize the need for pharmacokinetic and clinical bioequivalence trials, the generation of NPK must be multi-disciplinary. Steps needed to identify and characterize products, define knowledge gaps, and generate, validate, and peer-review NPK before its public dissemination would need to occur in time to support approval of “first generic” immediately after the expiration of the patent term and other exclusivity periods for the reference listed drug product. A multi-university collaboration, such as NIPTE, which, since its inception, has worked on resolving regulatory science problems and has worked to build infrastructure for knowledge management (see (1) and (4)) is ideally suited to take up the responsibility for generating, curating, and disseminating NPK to support the current system in more efficiently meeting the needs of the Nation.

The research effort needed to generate, assimilate, curate, and disseminate NPK will be aligned with NIPTE’s education and professional development certification programs. The NPK will be disseminated publicly in a manner (timing, content, and format) that can be utilized by industry and regulators to facilitate product development, regulatory review, and inspection efforts. Generic companies, for example, could utilize NPK to plan their development program to be more rigorous (in terms of risk assessment) and efficient (minimize additional studies and trials), eliminate multiple regulatory review cycles, more confidently demonstrate process controls, and prevent quality failures. Regulators can utilize NPK to ensure quality by design more confidently and be risk-based in their assessment of “Good Practices” in commercial operations to prevent quality failures and reduce the need for enforcement actions that generate negative media and social media coverage. The outcome NPK efforts should be objectively measurable in the real world, timely availability of competitive products that can be reliably manufactured to deliver high assurance of therapeutic equivalence that patients need and expect.

The proposed NIPTE model of Team Science for Pharmaceutical NPK (for example see (5) and (21)) serves as an example of a mature academic community of pharmaceutical knowledge capable of taking on increasing responsibility to establish a system to generate NPK on critical aspects of pharmaceutical formulation, product quality and performance, and failure modes of anticipated generic and biosimilar products.

NIPTE’s collective sense of urgency on NPK stems for the juxtaposition of the many and recurring contemporary challenges in the assurance of quality, the increasing mandates (*e*.*g*., 21st Century Cures Act), opportunities, and duty of care to take responsibility to be more patient-centric in what we do and to utilize “real world” evidence in our decision-making. The current level of funding, as is summarized in the first paper on the impact of NIPTE (33), is sorely inadequate to fill gaps in prior pharmaceutical knowledge which is essential to ensure and sustain availability and affordability of pharmaceuticals with the assurance of therapeutic equivalence US patients need in the twenty-first century.

Shortly after the formation of NIPTE, Senator Richard Lugar had introduced a legislative remedy to the challenges the proposed NPK seeks to address—Pharmaceutical Technology and Education Enhancement Act to the US Senate on 11 May 2006. The bill (S. 2793) sought to enhance research and education in the areas of pharmaceutical and biotechnology science and engineering, including therapy development and manufacturing, analytical technologies, modeling, and informatics (34). The need remains acute for a similar comprehensive remedy. NIPTE should now seek a similar legislating remedy to establish a system for NPK.
